# Supplementary material for: Adaptation to Nighttime Light via Gene Expression Regulation in Drosophila suzukii
Source: Ecol Evol. 2025 Aug 11;15(8):e71971. doi: 10.1002/ece3.71971 (PMC12339407; doi:10.1002/ece3.71971)
Supplement: Supplementary file 1 — Tables S1–S7: ece371971‐sup‐0001‐Tables.docx. [file ECE3-15-e71971-s001.docx]

# Supplemental Information

**Table S1.** Populations used in the experiments.

| Population | Latitude | Longitude | Urbanization type | Experiment | |
| --- | --- | --- | --- | --- | --- |
| ITA | 35.3206000 | 140.1423000 | rural | BS, LA, GE | |
| RES | 35.4784570 | 140.2439530 | rural | BS, LA, GE |  |
| INO | 35.7002000 | 139.5764000 | urban | BS, SR, LA, GE |  |
| SUM | 35.7126000 | 139.8040000 | urban | BS, SR, LA |  |
| YAY | 35.6287000 | 140.1026000 | urban | SR, LA |  |
| NOG | 35.4631000 | 140.1129000 | rural | SR, LA, GE | |
| KSI | 35.6407740 | 139.8553310 | urban | SR, LA | |
| KUR | 35.2877140 | 140.0898190 | rural | SR, LA | |
| TEN | 35.7056890 | 140.5477960 | rural | SR, LA | |
| TKB | 36.2206880 | 140.1051640 | rural | SR, LA | |
| WAK | 35.6755870 | 139.9936830 | urban | SR, LA | |
| ASU | 35.7504718 | 139.7366288 | urban | SR, LA, GE | |
| TTY | 34.9803920 | 139.8555450 | rural | SR, LA | |
| STB | 35.6936817 | 139.7385036 | urban | SR, LA, GE | |
| IZU | 35.5789340 | 140.2268830 | rural | LA | |
| SEA | 35.6093087 | 139.7474079 | urban | LA | |

BS, test for body size; SR, test for survival rate; LA, test for locomotor activity; GE, test for gene expression.

**Table S2.** Mean, minimum, maximum, and median of survival days.

| Urbanization type | lighting | sex | Mean | Minimum | Maximum | Median |
| --- | --- | --- | --- | --- | --- | --- |
| rural | 0 | female | 32.9 | 4 | 57 | 30 |
| rural | 0 | male | 40.6 | 24 | 69 | 32 |
| rural | 1 | female | 40.5 | 9 | 85 | 38 |
| rural | 1 | male | 35.4 | 3 | 72 | 35 |
| urban | 0 | female | 18 | 5 | 45 | 16 |
| urban | 0 | male | 25.9 | 3 | 81 | 25 |
| urban | 1 | female | 44.3 | 21 | 69 | 44.5 |
| urban | 1 | male | 28.5 | 5 | 66 | 21 |

**Table S3.** Results of the Cox mixed-effects model for the survival rate.

| Factors | *Z* | *P* |
| --- | --- | --- |
| light treatment | 0.61 | 0.54 |
| urbanization type | 0.57 | 0.57 |
| sex | 0.18 | 0.86 |
| light treatment × urbanization type | −2.17 | 0.03 |
| light treatment × sex | 0.36 | 0.72 |
| urbanization type × sex | 0.01 | 0.99 |
| light treatment × urbanization type × sex | 0.06 | 0.95 |

**Table S4.** Results of LMM for the PC1 valued of principal component analysis of gene expression of males.

| Factors | χ^2^ | *P* |
| --- | --- | --- |
| Light treatment | 0.044 | 0.83 |
| Urbanization type | 2.1 | 0.15 |
| Light treatment × urbanization type | 0.13 | 0.72 |

**Table S5.** Results of LMM for the PC1 values of PCA of gene expression in females.

| Factors | χ^2^ | *P* |
| --- | --- | --- |
| Light treatment | 13.0 | 0.00030 |
| Urbanization type | 1.9 | 0.17 |
| Light treatment × urbanization type | 3.1 | 0.078 |

**Table S6.** Results of LMM for the PC2 values of PCA of gene expression in males.

| Factors | χ^2^ | *P* |
| --- | --- | --- |
| Light treatment | 0.022 | 0.88 |
| Urbanization type | 0.45 | 0.50 |
| Light treatment × urbanization type | 1.2 | 0.28 |

**Table S7.** Results of LMM for the PC2 values of PCA of gene expression in females.

| Factors | χ^2^ | *P* |
| --- | --- | --- |
| Light treatment | 1.4 | 0.24 |
| Urbanization type | 0.44 | 0.51 |
| Light treatment × urbanization type | 4.9 | 0.026 |
